# Simultaneous Hydrodistillation-Steam Distillation of *Rosmarinus officinalis*, *Lavandula angustifolia* and *Citrus aurantium* from Morocco, Major Terpenes: Impact on Biological Activities

**DOI:** 10.3390/molecules26185452

**Published:** 2021-09-07

**Authors:** Sara El Kharraf, Maria Leonor Faleiro, Farah Abdellah, Soukaïna El-Guendouz, El Mestafa El Hadrami, Maria Graça Miguel

**Affiliations:** 1 Applied Organic Chemistry Laboratory, Faculty of Sciences and Technique, University Sidi Mohamed Ben Abdellah, BP: 2202, Imouzzer, Fes 30000, Morocco; sara_kh_17@hotmail.fr (S.E.K.); farah.abdellah1@gmail.com (F.A.); elmestafa.elhadrami@usmba.ac.ma (E.M.E.H.); 2 Faculdade de Ciências e Tecnologia, C8, Campus de Gambelas, Universidade do Algarve, 8005-139 Faro, Portugal; mfaleiro@ualg.pt (M.L.F.); soukaina.elguendouz@gmail.com (S.E.-G.); 3 Algarve Biomedical Center, Research Institute, 8005-139 Faro, Portugal; 4 Champalimaud Research Program, Champalimaud Centre for the Unknown, 1400-038 Lisbon, Portugal; 5 Mediterranean Institute for Agriculture, Environment and Development, Faculdade de Ciências e Tecnologia, C8, Campus de Gambelas, Universidade do Algarve, 8005-139 Faro, Portugal

**Keywords:** antioxidant activity, enzyme inhibitory activity, antimicrobial activity, anti-biofilm formation

## Abstract

Interest in the use of essential oils (EOs) in the biomedical and food industries have seen growing over the last decades due to their richness in bioactive compounds. The challenges in developing an EO extraction process that assure an efficient levels of monoterpenes with impact on biological activities have driven the present study, in which the EO extraction process of rosemary, lavender and citrus was performed by simultaneous hydrodistillation–steam distillation, and the influence of EO composition on biological activities, namely antioxidant, anti-inflammatory, antidiabetic, anti-acetylcholinesterase, anti-tyrosinase, antibacterial, and antibiofilm activity, were evaluated. The EO yields of combinations were generally higher than the individual plants (*R. officinalis* (*Ro*), *L. angustifolia* (*La*), and *C. aurantium* (*Ca*)) extracted by the conventional hydrodistillation. The EOs obtained by this process generally had a better capacity for scavenging the free radicals, inhibiting α-glucosidase, and acetylcholinesterase activities than the individual EOs. The combination of EOs did not improve the ability for scavenging peroxide hydrogen or the capacity for inhibiting lipoxygenase activity. The antioxidant activity or the enzyme inhibition activity could not only be attributed to their major compounds because they presented lower activities than the EOs. The chemical composition of the combination *Ro*:*La*:*Ca*, at the ratio 1/6:1/6:2/3, was enriched in 1,8-cineole, linalool, and linalyl acetate and resulted in lower MIC values for all tested strains in comparison with the ratio 1/6:2/3:1/6 that was deprived on those components. The biofilm formation of Gram positive and Gram negative bacteria was impaired by the combination *Ro*:*La*:*Ca* at a sub-inhibitory concentration.

## 1. Introduction

Oxidative stress can lead to the initiation of pathological mechanisms, which leads to cell damage and consequently to the development of many pathogenic diseases such as diabetes and other metabolic disorders, Alzheimer’s, skin cancer, and cardiovascular diseases, either by stimulating the generation of reactive oxygen species (ROS) and reactive nitrogen species (RNS) at abnormally high concentrations, or by a decline in cell antioxidant defence mechanisms [1]. Moreover, oxidative stress can also be triggered by the presence of bacterial infections, which together with the increase and rapid spread of resistant pathogens, such as the methicillin-resistant *Staphylococcus aureus* (MRSA) strains, is a major public health concern [2,3].

*Staphylococcus aureus* (Gram positive) and *Escherichia coli* (Gram negative) are two commensal bacteria that can became opportunistic foodborne pathogens, but are also responsible for post-operative wound infection, and represent a frightening threat in the hospitals and food industries [4,5]. Furthermore, bacterial cells that adhere to an abiotic or biotic surface are able to produce a polymeric matrix that allows the sessile cells (adherent) to become 10–1000 times more resistant in comparison with planktonic cells (in suspension), acquiring the ability to overcome the presence of antimicrobial agents and disinfectant products being responsible for 75% of infections and contaminations that are mainly associated with contaminated medical devices, water distribution systems, and the food industry [6,7,8].

In recent years, essential oils (EOs) have been exploited as natural antioxidants and antimicrobial agents due to a number of highly active compounds, such as monoterpenes and sesquiterpenes (oxygenated or not) [9,10]. Many in vitro, in vivo, and epidemiological researches have been carried out on EOs and their main compounds, such as thymol, carvacrol, linalool, camphor, limonene, carvone, carvacrol, and general terpenes, under different pathological oxidative stress situations, as well as in the case of nosocomial infections and foodborne disease [11,12,13].

Rosemary (*Rosmarinus officinalis* L., Lamiaceae (*Ro*)) and lavender (*Lavandula angustifolia* Mill., Lamiaceae (*La*)) are species distributed along the Mediterranean region and are widely cultivated due to their biological properties (choleretic, hepatoprotective, a stimulant, memory improvement, antioxidant, antimicrobial) [14,15,16,17,18] with applications in food, pharmaceutical and cosmetic industries [14,15]. They have been used since ancient times in traditional medicine and for flavoring foods. Usually, EOs are extracted from the leaves of *Ro* and the flowers of *La*. The EOs of *Ro* are mainly constituted of α-pinene, camphene, β-pinene, 1,8-cineole, camphor, and borneol, the percentages of which vary, originating from two main chemotypes: Spanish and Moroccan, and Tunisian types [14]. The EOs of *La* are predominantly constituted of linalool, lavandulol, and linalyl acetate [15].

Citrus (*Citrus* × *aurantium* L., Rutaceae (*Ca*)), bitter orange, is a citrus tree originally distributed in South East Asia. It was principally used in traditional Chinese medicine as a booster for vital energy and circulation. Moreover, numerous studies have investigated the antioxidant, antimicrobial, antifungal, anti-diabetic, and anti-inflammatory capacities of its essential oils and extracts from peels and flowers [16,17,18,19]. In addition, this species has been also applied in the treatment of prostate cancers [20], anxiety [21], gastrointestinal diseases, and obesity [16,22]. Several studies have been focused on the pharmaceutical, cosmetic, and food applications of different parts of *C. aurantium*, such as fruits and flowers, and much less with the leaves which EOs are mainly constituted of in linalool, terpinen-4-ol, limonene, neryl acetate, and linalyl acetate [23].

The present work aims to investigate the influence of the simultaneous hydrodistillation–steam distillation of the combinations *Ro:La*; *Ro:Ca*; *Ca:La*; *Ro:La:Ca* on EO compositions and their biological properties, particularly antioxidant, anti-inflammatory, antidiabetic, anti-acetylcholinesterase, anti-tyrosinase, and antibacterial activity, against methicillin-susceptible *S. aureus*, methicillin-resistant *S. aureus* (MRSA), and *E. coli*, as well as their capacities to prevent bacterial biofilm formation.

## 2. Results and Discussion

### 2.1. Yields and Chemical Composition of Essential Oils Extracted by a Hydrodistillation and a Simultaneous Hydrodistillation–Steam Distillation

In the present study, the EOs tested were of the same species and were extracted in the same conditions (hydrodistillation and simultaneous hydrodistillation–steam distillation) of those previously reported by El-Kharraf et al. [24], but were collected one year later, in order to obtain enough material for the determination of the biological properties.

**Figure 1:**
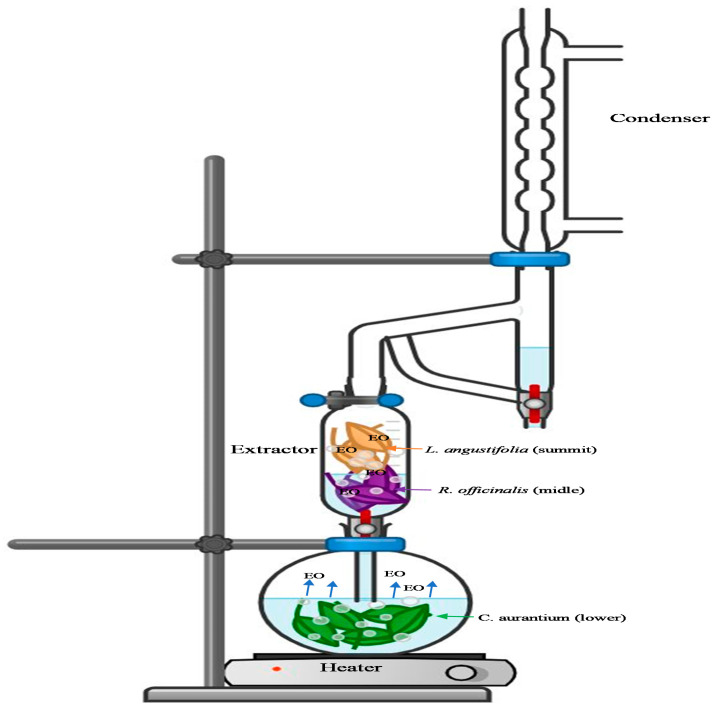
Simultaneous hydrodistillation–steam distillation apparatus, as described in [24].

The scheme of the EO extraction in combination using a simultaneous hydrodistillation–steam distillation is illustrated in Figure 1. The EO yields and organoleptic characteristics of *Ro*, *La*, and *Ca*, and their combination values, are presented in Table 1.

**Figure 2:**
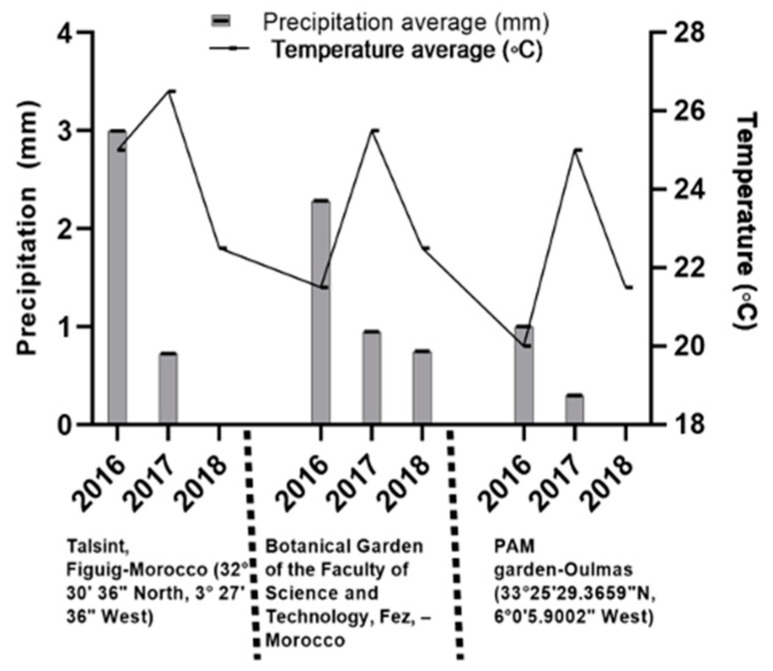
*Variation* of climatic conditions in the collection regions of *Ro*, *La*, and *Ca* between June 2016 and June 2018. Data are collected from: https://www.meteoblue.com/fr/meteo/historyclimate/weatherarchive/figuig_maroc_6546275 (Accessed date: 15 June 2021).

The *Ro* and *La* oils were colorless, with a heavy woodsy smell and floral–herbaceous ordor, respectively. While *Ca* oil was a little greenish with an herbaceous smell and a slightly sweet aroma at the end. Notably, the sample combinations had slightly different organoleptic characteristics as single EOs, which varied depending on the plant ratio in the mixture; for example, *Ro*:*La*:*Ca* (1:1:1) had a light yellowish color with an herbaceous and strong citrus smell (Table 1). Moreover, the results show that the EO yields of the sample combinations were significantly higher and ranged from 1.35% to 2.26% (mL/100 g of plants) compared to the individual plants (*Ro*, *La*, and *Ca*) extracted by conventional hydrodistillation (1.50%, 1.23%, and 0.76%, mL/100 g of plants). These results are somehow different to those observed previously, which can be partially explained by the climatic conditions, which may influence the production of EOs. The plants were collected at the same locations as the previous work [24]. The climatic conditions observed during the period 2016 to 2018 underwent an appreciable modification, with an accentuated diminution of precipitation (Figure 2). For other species, including *Ro*, Mehalaine, and Chenchouni [25] it was found that the precipitation had a positive effect on the accumulation of EOs. 

The binary combination *Ro*:*La* sample yielded 2.26 ± 0.04% mL/100 g of plants (*p*-value < 0.001), a yield higher than the tertiary combination samples obtained by simultaneous hydrodistillation–steam distillation, where similar yields values (1.50–1.65% mL/100 g of plants) were observed (Table 1). 

The main compounds with percentages higher than 5%, present in the EOs obtained by hydrodistillation and simultaneous hydrodistillation–steam distillation, are summarized in Table 2. The EO of the *Ro* sample contains, in the majority, one compound-1,8-cineole (46.71%)-followed by α-pinene (13.83%) and camphor (13.07%). This EO is a Moroccan and Tunisian chemotype, according to the European Pharmacopoeia [26]. In the EO of *La*, the major compounds were linalool (21.34%), camphor (14.18%), 1,8-cineole (13.9%), borneol (11.77%), and linalyl acetate (11.58%), which is in accordance with previous studies [27,28] on Moroccan and Turkish lavender EOs. The samples of *Ca* EOs had as major compounds linalyl acetate (36.00%), and linalool (30.77%), followed by α-terpineol (14.97%).

The sample *Ca:La* contained linalyl acetate (23.54%) and linalool (19.33%) as major components, followed by camphor (11.36%) and 1,8-cineole (11.14%), while borneol and α-terpineol were in lower amounts in comparison with *Ca* and *La* EOs. The 1,8-cineole and camphor percentages were similar in both combinations *Ro:La* and *Ro:Ca* EOs; on the other hand, the linalyl acetate percentage in the two samples was inferior to 10% and reached 10.71%, respectively.

The tertiary combination *Ro:La:Ca* (1:1:1) contained 1,8-cineole (27.77%), linalool (12.99%), camphor (12.65%), and linalyl acetate (11.20%); α-pinene, borneol, and α-terpineol were also detected in small amounts, despite the fact that they were present in the individual oil in a relative high amount (higher than 10%). 1,8-Cineole, linalool, and camphor percentages were essentially similar to the theoretical values of combined *Ro*, *La*, and *Ca* EOs in the ratios (1:1:1) (Table 2); however, the percentage of linalyl acetate in the combination was lower than the theoretical amount. The major components detected in the samples *Ro:La:Ca* (2/3:1/6:1/6), (1/6:2/3:1/6), and (1/6:1/6:2/3) were similar to those present in the individual EOs. However, the result showed that the component percentages estimated in those samples were higher in comparison with the theoretical values, where each sample has to contain only 66.67% of the component of the plants with a higher ratio in the mixture (2/3 ratio). In general, during the simultaneous hydrodistillation–steam distillation, the steam water and essential vapor from the *Ca* plant in the lower unit of the Clevenger apparatus penetrated the *La* (middle) and *Ro* (summit) in the extractor, which generated a better extraction condition in some compounds of the EOs. It seems evident that the EO yields, and the concentration of volatile compounds are noticeably influenced by plant ratio in the combination using the simultaneous hydrodistillation–steam distillation system.

### 2.2. Antioxidant Activity

**Figure 3:**
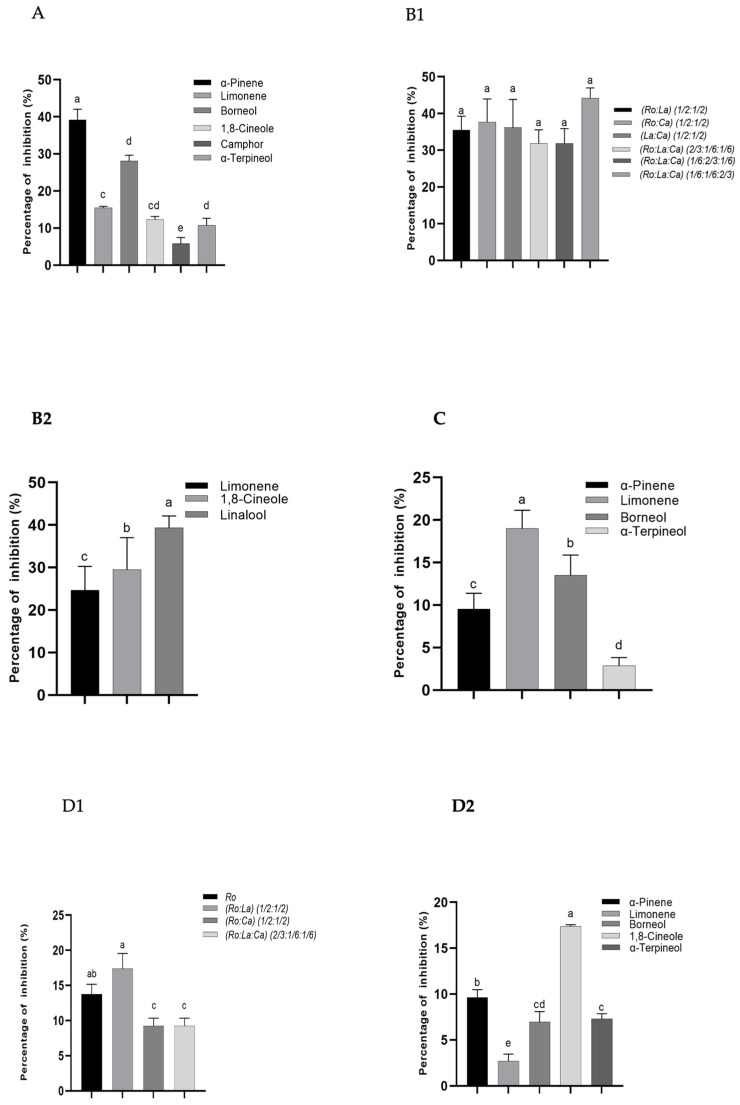
Antioxidant activities. (**A**) Percentage of 2,2-diphenyl-1-picrylhydrazyl (DPPH) free radical-scavenging activity of the major components: α-pinene (215 mg/mL), limonene (4 mg/mL), borneol (4 mg/mL), 1,8-cineole (231 mg/mL), camphor (500 mg/mL), α-terpineol (209 mg/mL), (**B1**) Percentage of H_2_O_2_ inhibition for *Ro* (45 mg/mL)*, La* (42 mg/mL)*, Ca* (42 mg/mL)*,* (*Ro:La)* (46 mg/mL)*,* (*Ro:Ca*) (43 mg/mL), (*La:Ca*) (46 mg/mL), (*Ro:La:Ca*) (1:1:1) (42 mg/mL), (*Ro:La:Ca*) (2/3:1/6:1/6) (43 mg/mL), (*Ro:La:Ca*) (1/6:2/3:1/6) (42 mg/mL), (*Ro:La:Ca*) (1/6:1/6:2/3) (42 mg/mL), (**B2**) Percentage of H_2_O_2_ scavenging of the major components (limonene (4 mg/mL), 1,8-cineole (5 mg/mL), linalool (4 mg/mL), (**C**) Percentage of nitric oxide free radical-scavenging of the major volatiles α-pinene (9 mg/mL), limonene (4 mg/mL), borneol (4 mg/mL), α-terpineol (5 mg/mL), (**D1**) Percentage of superoxide anion scavenging for *Ro* (15 mg/mL), (*Ro:La*) (8 mg/mL), (*Ro:Ca)* (7 mg/mL), and (*Ro:La:Ca*) (2/3:1/6:1/6) (15 mg/mL); (**D2**) Percentage of superoxide anion scavenging of the major components α-pinene (1 mg/mL), limonene (8 mg/mL), borneol (8 mg/mL), 1,8-cienole (8 mg/mL), α-terpineol (8 mg/mL). Bars represent standard deviations (n = 3). Values with the same letter are not significantly different (*p* > 0.05) by Tukey’s multiple range test.

The antioxidant abilities of the EOs obtained by hydrodistillation and simultaneous hydrodistillation–steam distillation and their major compounds were evaluated through different assays, namely free radical scavenging activities and hydrogen peroxide scavenging activity. The results are depicted in Table 3, for those samples that permitted to determine IC_50_ values (concentrations providing 50% inhibition). For the remaining, where it was not possible due to their weak activities or interferences with relative high concentrations, the results are presented in inhibition percentages and are depicted in Figure 3A–D.

#### 2.2.1. 2,2-Diphenyl-1-Picrylhydrazyl (DPPH) Free Radical-Scavenging Activity

The concentrations of EOs from the individual plants and their combinations able to scavenge 50% of free radical are presented in Table 3. The combinations *Ro:Ca*, *La*:*Ca*, and *Ro:La:Ca* (1/6:1/6:2/3) exhibited the highest inhibition effects, with an IC_50_ inferior to 1 mg/mL, but were higher than of that of the positive control butylated hydroxytoluene (BHT) [29]. The activities found cannot be attributed to the single compounds present in the EOs since their IC_50_ values were higher than the EOs. Only for linalool and linalyl acetate (102.34 and 148.61 mg/mL) was it possible to determine the IC_50_ values. For the remaining terpenes, it was only possible to evaluate the inhibition percentage for a specific concentration (Figure 3A). Regarding the single EOs, the *Ca* EO was the best one, in which, when in association with *Ro* EO, the free radical scavenging activity became even enhanced (Table 3). Perhaps it was the case that the association of camphor and 1,8-cineole (*Ro*) with linalool, linalyl acetate (*Ca*) and other minor components produced a synergistic effect.

#### 2.2.2. Hydrogen Peroxide (H_2_O_2_) Scavenging Activity

Overall, all samples poorly scavenged H_2_O_2_. The capacity for scavenging H_2_O_2_ was not significantly different (*p* < 0.05) among those samples where IC_50_ values were possible to determine (Table 3). They were much lower, at least 6500 times less active, than the positive control (ascorbic acid) [29]. The remaining EOs extracted by simultaneous hydrodistillation–steam distillation had a very weak inhibitor capacity and they barely scavenged 26% to 40% of H_2_O_2_ for a concentration equal to 46 mg/mL, as shown in Figure 3B1. Only two terpenoids (1,8-cineole and linalool) and one terpene (α-pinene) had some ability to scavenge H_2_O_2_, at least for the highest concentration that was permitted to evaluate the activity (Figure 3B2). 

Hydrogen peroxide is a non-radical oxygen species with a high capacity for crossing through cell membranes and inside the cells can react with transition metals giving rise hydroxyl radicals, where at high concentrations these radicals induce peroxidation of lipids and proteins with harmful effects on the cell integrity. For this reason, it is important to find compounds able to scavenge H_2_O_2_ [30]. The results obtained in the present work reveal that the EOs of the three aromatic plants and their blends obtained by the simultaneous hydrodistillation–steam distillation are not able to scavenge H_2_O_2_ efficiently. However, these results may also be attributed to the reaction media; that is, the reaction is made in an aqueous media and the EOs are hydrophobic natural products.

#### 2.2.3. Nitric Oxide Free Radical Scavenging Activity

The best nitric oxide radical scavenging capacity was detected for the *Ca* EO (Table 3) and the IC_50_ value estimated was closer to the chosen positive control curcumin (0.25 mg/mL and 0.01 mg/mL, respectively) [29] than the remaining EOs, or their major compounds. The IC_50_ values found for *Ca* EO was followed by those of *Ro*:*La*:*Ca* (1/6:1/6:2/3) (1.12 mg/mL) and *La*:*Ca* (1/2:1/2) (1.56 mg/mL). These observations may illustrate the importance of the citrus EO on the activity. No studies were reported concerning the extraction of *Ca* EO from leaves; nevertheless, Peana et al. [31] reported that the linalool prevented the formation of the nitric oxide radical. In the present work, the formation of nitric oxide was not studied, but the capacity for scavenging these radicals, and in this case linalool and linalyl acetate, had no ability (Table 3). Therefore, a synergistic effect may occur among the several compounds present in the EOs, which always makes it difficult to foresee the biological activities of these natural compounds and consequent medical application. Considering individual volatile compounds, only very few (α-pinene, limonene, borneol and α-terpineol) had activity (Figure 3C) at the concentrations permitted to evaluate this activity.

#### 2.2.4. Superoxide Anion Radical Scavenging Activity

Superoxide anions radical scavenging activity was significantly higher for samples *Ro:La:Ca* (1/6:1/6:2/3) (IC_50_ = 1.39 mg/mL), yet were still inferior to the positive control l-ascorbic acid activity [29], succeeded by less effective samples, such as *Ro* [29], *Ca*, *Ro:La:Ca* (1/6:2/3:1/6), and *La*:*Ca* (1/2:1/2) within IC_50_ values higher than 2 mg/mL but lower than 3.5 mg/mL, while the IC_50_ value for the *Ro:La:Ca* (1/3:1/3:1/3) EO was higher than 10 mg/mL (Table 3). The remaining samples (*Ro*, *Ro:La*, *Ro:Ca*, and *Ro:La:Ca* (2/3:1/6:1/6)) had a weak superoxide anion scavenging effect for a final concentration of 7–15 mg/mL that scavenged only 4% to 17% of the free radicals (Figure 3D1).

The results reported by Aazza et al. [32] for *Ro* and *La* EOs were similar to those found in the present work and were lower compared with those reported by Parejo et al. [33] No reports on scavenging superoxide anions radical activity of EOs obtained from *Ca* leaves were found. Some authors [34,35,36] reported that α-pinene, α-terpineol, linalool, 1,8-cineole, and linalyl acetate had a moderate inhibitory capacity toward superoxide anions radicals, while camphor had no activity, these results being similar to those found in the present work and depicted in Table 3 and Figure 3D2. A negative correlation can be observed between the amount of the *Ca* EO in the samples and the IC_50_ values, being the EO of *Ca* with the best capacity for scavenging the superoxide anion radicals, i.e., increasing the *Ca* ratio in the samples corresponds to high free radical scavenging activities. 

*Ro:La:Ca* (1/6:1/6:2/3) and *Ro:La:Ca* (1/3:1/3:1/3) showed the highest scavenging nitric oxide radicals and superoxide anion radicals activities, inhibiting, therefore, the formation of peroxynitrite and hydroxyl radicals, respectively and being responsible for the oxidation of the biomolecules, which causes damage to cells. On the contrary, it was noticed that the high amount of rosemary in the EOs decreased their activities. 

### 2.3. Enzymatic Activity 

#### 2.3.1. Anti-Glucosidase Activity

The in vitro enzymatic inhibitory assays of EOs extracted by hydrodistillation, simultaneous hydrodistillation–steam distillation, and major components as well as positive controls are depicted in Table 4.

Dietary carbohydrate is metabolized in the human body by several steps, ending in α-glucosidase activity located in intestinal cells, which catalyse the hydrolysis of carbohydrates to monosaccharides, the absorption which causes the increased blood glucose levels [37]. The inhibition of the enzyme could restrain hyperglycaemic episode by retarding the carbohydrates degradation and their migration to blood vessels. The samples *La*, *Ro:La*, and *Ro:La:Ca* (1/6:2/3:1/6) are reported to have a remarkable inhibitory capacity (IC_50_ = 0.05 mg/mL), which is not significantly different (*p* < 0.05) to the acarbose IC_50_ value. Those samples had, as major volatile compounds, 1,8-cineole, camphor, borneol, linalool, and linalyl acetate. However, when analysed separately, these volatile components showed a low inhibitory activity against α-glucosidase, and linalool and linalyl acetate did not have activity (Table 4). The results only can be explained by a synergetic effect among the compounds of the EOs. The samples *Ca*, *Ro:Ca* and *Ro:La* possessed a good inhibitory effect (0.16 mg/mL, 0.11 mg/mL and 0.08 mg/mL, respectively). It is worth noting the importance of La EOs on the inhibitory activity of α-glucosidase, despite the absence of activity of their main components, linalool, linalyl acetate, and α-terpineol, which had no activity. The remaining samples (Ro:La:Ca (1/3:1/3:1/3)) had a moderate activity (IC_50_ = 0.36 mg/mL), immediately followed by Ro, α-pinene, camphor, *Ro:La:Ca* (1/6:1/6:2/3), limonene, borneol, and 1,8-cineole. The IC_50_ values for *Ro* EO was inferior to that of found by Ahamad et al. [38] on the rosemary from Iraq, the main components of which were verbenone, 1,8-cineole, and α-pinene. Nonetheless, the *La* EO on α-glucosidase inhibitory activity, evaluated in the present work, was better than that mentioned by Dhasthakeer et al. [39] The *Ca* EO had a similar inhibition activity to the one evaluated by Dang et al. [40], with the IC_50_ value of 0.41 mg/mL, but was obtained from peels predominantly constituted of limonene.

#### 2.3.2. Anti-Acetylcholinesterase Activity

A strategy for retarding the decline in cognitive abilities and memories in Alzheimer’s disease is to use medicines that act on acetylcholinesterase (AChE) activity, inhibiting it [37]. Several natural agents as essential oils have been investigated for their potential inhibitory capacities. In the present work, the sample *Ro:La:Ca* (2/3:1/6:1/6) had the lowest IC_50_ value (0.03 mg/mL) and the closest to the positive control, galantamine (IC_50_ = 0.001 mg/mL) [29]. The samples *Ro:La*, *Ro:Ca* and *La*:*Ca* had good enzymatic inhibitor effects (IC_50_ = 0.05 mg/mL, 0.07 mg/mL and 0.06 mg/mL, respectively), followed by the remaining tertiary combined EOs *Ro:La:Ca* (1/3:1/3:1/3) with IC_50_ = 0.08 mg/mL; poorer activity was measured for the individual EOs Ro [29], *La*, and *Ca*, with IC_50_ = 0.34, 0.44, and 2.87 mg/mL, respectively. Limonene and 1,8-cineole had good inhibitory activities when compared with the other terpenes (IC_50_ = 0.08 and 0.18 mg/mL, respectively). As aforementioned for other assays, the role of a single volatile compound is less important when compared with EOs, or even blended EOs. In this case, blended EOs were able to act as inhibiting acetylcholinesterase activity much better than the single EOs or their volatile constituents. 

Concerning individual volatile compounds, Movahhedin et al. [41] reported that 1,8 cineole was a potent acetylcholinesterase inhibitor. In the present work, this volatile had the capacity for inhibiting the enzyme, but when in association with other volatiles in the EOs it may act synergistically, leading to better activities (Table 2 and Table 4). The IC_50_ value of Ro [29] (0.34 mg/mL) was similar to that reported by some authors [42,43] for Turkish and Spanish *Ro* with major compounds such as 1,8-cineole, camphor, and α-pinene. As for the *La* inhibitory ability was greater than those previously reported by Ferreira et al. [44] where only 39.5% of enzyme inhibition could be observed with 1 mg/mL of lavender oil. In addition, Dohi et al. [45] mentioned that the major compounds in *La* EO were 1,8-cineole, α-pinene, and α-terpineol that exhibited a suitable potential against acetylcholinesterase (IC_50_ = 0.01, 0.02, and 1.3 mg/mL, respectively). However, no work had been reported in the activity of *Ca* EO extracted from the leaves by hydrodistillation toward acetylcholinesterase, besides the work carried out by Loizzo et al. [46] on the n-hexane extract of *Ca* leaves, which has shown a remarkable activity (IC_50_ = 0.09 mg/mL) due to the presence of limonene.

#### 2.3.3. Anti-Lipoxygenase Activity

To estimate the anti-inflammatory activity, the linoleic acid was used instead of arachidonic acid, a precursor in the biosynthesis of the proinflammatory leukotrienes [37]. Ca EO and limonene had similar IC_50_ values and exhibited a good anti-5-lipoxygenase ability compared with the other samples, but remained inferior to the positive control nordihydroguaiaretic acid (NGDA) [29]. The presence of limonene in citrus EO might be responsible for its anti-enzymatic activity; on the other hand, the oxygenated monoterpenes, such as linalool, camphor, 1,8-cineole, and α-terpineol, displayed weak anti-inflammatory activity (Table 4). Similar results were observed by Frum et al. [47], who explained that limonene activity is probably produced by its nonpolar structure, which allows it to reach the enzyme active site. The oxygenated monoterpenes could not cleave the 5-lipoxygenase due to electronegative repulsion between the electronegative nitrogen atom places in the enzyme site and the hydroxyl function of oxygenated monoterpenes. However, the amount of limonene is very low in the EO of *Ca*, linalool and linalyl acetate being the most important monoterpenoids. In this case, linalyl acetate also had inhibitory activity on lipoxygenase, which may be partly explained by the replacement of the hydroxyl group of linalool by an acetate group (ester), probably with a lower electronegative repulsion between the functional groups of the monoterpenoid and the enzyme active site.

**Figure 4:**
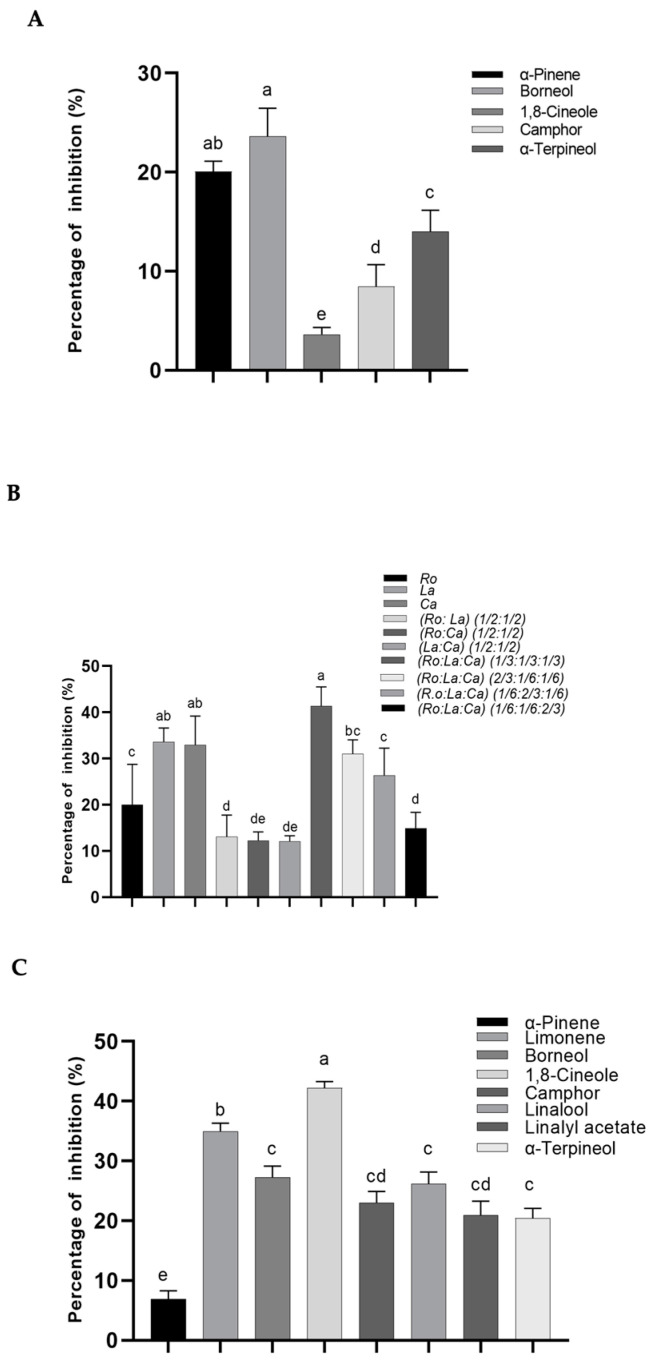
Enzymatic activities. (**A**) Percentage of lipoxygenase enzyme inhibition of the major components α-pinene (1 mg/mL), borneol (1 mg/mL), 1,8-cienole (1 mg/mL), camphor (1 mg/mL), α-terpineol (1 mg/mL); (**B**) Percentage of tyrosinase enzyme inhibition obtained from EOs of samples (4.0–4.6 mg/mL) extracted by hydrodistillation and simultaneous hydrodistillation–steam distillation; (**C**) Percentage of tyrosinase enzyme inhibition of the major components (50 mg/mL). Bars represent standard deviations (n = 3). Values with the same letter are not significantly different (*p* > 0.05) by Tukey’s multiple range test.

The poorest anti-inflammatory action was estimated for *Ro* (IC_50_ = 0.54 mg/mL) [29]. This poor effect may be partially explained by its major compound, such as 1,8-cineole [47]. In fact, this monoterpenoid presented the lowest activity (Figure 4A), in contrast to borneol. The IC_50_ value of rosemary oil was inferior to those found by other authors [17,43,48], but was higher than that reported by Aazza et al. [49] for the *Ca* leaf EOs from Rabat, Morocco (IC_50_ = 0.94 mg/mL). No previous works were reported on the EOs of *La*, although few reports had detailed the lipoxygenase inhibition of other species of lavender, such as *L. stoechas* L., in which an inhibition percentage of 29% for a concentration of 0.3 μL/mL in Spanish *L. stoechas* L. EO, was predominantly constituted of fenchone, 1,8-cineole and camphor [50]. In the present work, which concerns the major compounds of the EOs, limonene was presented as having the best ability to inhibit lipoxygenase activity (Table 4). The EO with the best activity was that of *Ca* where limonene was at a high percentage, although it was not the major one (Table 2). In addition, the remaining EOs with higher limonene percentages, but lower than citrus EOs, did not correspond to better activities (Table 4). Moreover, after limonene, borneol presented the best activity (Figure 4A). Nevertheless, it did not correspond to the best activity of *La* and *Ro* EOs or the *Ro:La* (1/2:1/2), where borneol was present at higher concentrations, confirming the importance of synergistic/antagonism effects among the volatile constituents of the EOs on the inhibitory lipoxygenase activity.

#### 2.3.4. Anti-Tyrosinase Activity

The tyrosinase inhibition percentages of EOs and their single major volatiles are depicted in Figure 4B,C. In any sample for which it was not possible to determine the IC_50_ values, only inhibition percentages for specific concentrations were determined and these are represented in Figure 4B,C. The samples *La*, *Ca*, *Ro:La:Ca* (1/3:1/3:1/3) and *Ro:La:Ca* (1/6:1/6:2/3) had a percentage of inhibition superior to 20% at the final concentration of 8 mg/mL (Figure 4B). Their major terpenes, such as 1,8-cineole > limonene > borneol, linalool > linalyl acetate, camphor, and α-terpineol, also exhibited an inhibitory effect (inhibition percentage > 20%), but at 50 mg/mL, and therefore displayed a poorer activity. The lowest ability was found for α-pinene. Those results might suggest a potential additive or/and synergistic effects, as aforementioned in the other assays. Meanwhile, the remaining samples—*Ro*, *Ro:La*, *Ro:Ca*, *La*:*Ca*, *Ro:La:Ca* (2/3:1/6:1/6), and *Ro:La:Ca* (1/6:1/6:2/3)—presented poorer activities against the enzyme with an inhibition percentage ranged between 12–20%, rather than the remaining EOs (Figure 4B). 

Aumeeruddy-Elalfi et al. [51] found that *Ro* EOs from Mauritius expressed good action against tyrosinase, and it could inhibit 50% of the enzyme at a final concentration of 0.09 mg/mL. This value was superior to the one previously ascribed in this work and the difference may be caused by the different chemical compositions in which limonene was present in the *Ro* EO from Mauritius and was absent for the one from Morocco. In contrast, the *Ro* EO from Yemeni was practically inactive, at only 3.3% inhibition at 0.10 mg/mL [52]. According to several authors [53,54,55] the *La* EO presented an important dose-dependent inhibitory effect on mushroom tyrosinase.

Matsuura et al. [56] reported that the capacity of inhibiting the enzyme was dependent on the citrus variety from Japan and could be attributed to the presence of myrcene geranial and neral. *Citrus grandis* EO, predominantly constituted by limonene, had a lower IC_50_ value than the positive control (kojic acid); that is, it had a better activity [51]. However, in the present work, limonene did not have the ability to inhibit lipoxygenase activity, at least in the assay conditions (Table 4).

### 2.4. Antibacterial Activity

Taking into account the good antioxidant and anti-enzymatic capacity of the combination *Ro:La:Ca*, which is rich in oxygenated monoterpenes, such as 1,8-cineole and linalool (Table 2, Table 3 and Table 4), its antibacterial activity was examined by an agar diffusion assay and the Minimum Inhibitory Concentration (MIC) and Minimum Bactericidal Concentration (MBC) values were determined. The results are summarized in Table 5. The highest inhibition zone was observed for *S. aureus* ATCC 6538 (24.00 ± 0.70 mm), followed by the MRSA 15 (18.33 ± 1.24 mm) at the ratio 1/6:1/6:2/3. At all ratios tested, the inhibition zones displayed by *E. coli* DSM 1077 were lower in comparison with the ones showed by *S. aureus* strains, suggesting a lower susceptibility of the Gram negative strain. The highest susceptibility to individual components was observed for linalool against *E. coli* DSM 1077 (15.67 ± 0.23 mm), followed by MRSA 15 and *S. aureus* ATCC 6538 (13.83 ± 1.24 and 12.83 ± 0.47 mm, respectively). Regarding the susceptibility of the antibiotic chloramphenicol (30 μm/mL), the *S. aureus* ATCC 6538 showed a similar inhibition zone (10.16 ± 0.70 mm) to the individual components 1,8-cineole and camphor (10.5 ± 0.70 and 10.5 ± 1.22 mm, respectively). The lowest inhibition zone for chloramphenicol was displayed by MRSA 15 (6.00 ± 0.00 mm). As expected, no inhibition zone was observed for 2-propanol (control). 

To the best of our knowledge, there are no reports on the synergistic antibacterial activity of the EOs of *Ro*, *La*, and *Ca*. In a previous study, linalool showed a higher inhibition zone (13.0 ± 4.2 mm) against *S. aureus* in comparison with *E. coli* (10.3 ± 0.5 mm), and the combination linalool:1,8-cineole (1:1) resulted in antagonism (lower inhibition zone of the combination in comparison with individual components) for both bacteria [57]. Such differences between the studies may be related to the different susceptibility of the used bacterial strains.

Similar MIC values for the ratio (1/6:2/3:1/6) were observed for *S. aureus* ATCC 6538 and *E. coli* DSM 1077 (10 μL/mL), in contrast with MRSA 15, which showed an MIC value 7.5 times higher (75 μL/mL). The MBC value for this ratio was also similar for *S. aureus* ATCC 6538 and *E. coli* DSM 1077 (25 mg/mL), whereas for MRSA 15 the MBC value was five times higher (125 μL/mL). The MIC values for the ratio 1/6:1/6:2/3 were <10 μL/mL for the three tested strains, and the MBC value was also <10 μL/mL for *S. aureus* ATCC 6538 and *E. coli* DSM 1077, but for MRSA 15 this value was equal to 10 μL/mL.

The higher MIC value of the ratio (1/6:2/3:1/6) may be associated with the low content of oxygenated monoterpenes, such as linalool and 1,8-cineole, and eventually the presence of camphor that, in the combination, may confer an antagonistic effect (Table 2). The chemical composition of the ratio 1/6:1/6:2/3 enriched in 1,8-cineole, linalool, and linalyl acetate resulted in lower MIC values for all tested strains. The individual use of 1,8-cineole, linalool, and linalyl acetate against MRSA strains was recently reported, and from the three monoterpenes, the lowest MIC value was displayed by linalool (2.83 ± 0.98 to 6.8 ± 1.0 μL/mL) followed by 1,8-cineole (57.56 ± 0.00 to 307.00 ± 132.93 μL/mL) and linalyl acetate (450.50 ± 0.00 μL/mL) [58]. It is not possible to discard the possible synergistic effect of other components that are present at low concentrations in the ratio (1/6:1/6:2/3). Nevertheless, the combination *Ro:La:Ca* seems to be very promising to combat MRSA strains and other resistant bacterial pathogens.

### 2.5. Antibiofilm Activity

**Figure 5:**
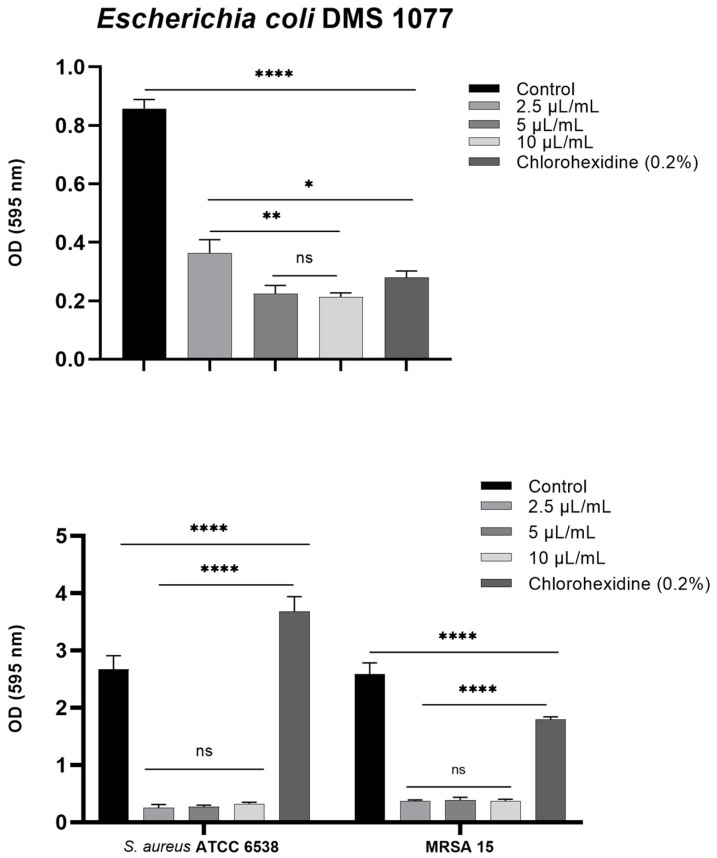
Impact of the combination *Ro:La:Ca* at ratio 0.16:0.16:0.67 on bacterial biofilm formation. Data are the mean of three independent experiments (n = 12). Error bars represent the standard deviation. * *p* < 0.05; ** *p* < 0.01; **** *p* < 0.0001, ns—not significant.

The impact of the combination *Ro:La:Ca* at the ratio 1/6:1/6:2/3 on the biofilm formation of *S. aureus* ATCC 6538 and *E. coli* DSM 1077 and MRSA 15 was evaluated, and the results are illustrated in Figure 5. The biofilm formation of the three tested bacteria was significantly impaired (*p* < 0.001) at all the concentrations tested (2.5 μL/mL, 5 μL/mL and 10 μL/mL) in comparison with the control of bacterial culture. However, the three tested concentrations were equally able (*p* > 0.05) to inhibit the biofilm of *S. aureus* ATCC 6538 and MRSA 15. In contrast to *E. coli* DSM 1077, it was noticed that the concentration 2.5 μL/mL caused a lower biofilm inhibition (*p* < 0.05) in comparison with the concentrations 5 and 10 μL/mL, which similarly (*p* > 0.05) inhibited the biofilm formation (Figure 5). The richness of the tested combination in 1,8-cineole, linalool, and linalyl acetate can explain the successful antibiofilm activity observed against both the Gram negative *E. coli* and the Gram positive *S. aureus* and the resistant MRSA 15. The ability of these components to successfully affect biofilm formation of pathogenic bacteria have been reported [59,60,61,62,63,64]. Interesting, chlorohexidine (0.2%, *v*/*v*) (positive control) induced the biofilm formation of *S. aureus* ATCC 6538, in contrast to *E. coli* DSM 1077 and MRSA 15, for which a reduced biofilm formation was observed (Figure 5). The failure of chlorohexidine (0.2%, *v*/*v*) to inhibit the adherence of *S. aureus* ATCC 6538 has been reported [65]. Interestingly, it was reported that the combination of 1,8-cineloe with chlorohexidine resulted in a synergistic effect against *S. aureus* and MRSA, both in planctonic (cells in suspension) and in biofilm [59,66].

EOs and their components may act against bacterial biofilms by disrupting the cell–cell communication system called quorum sensing (QS) [67,68,69,70]. The QS system is based on the production of signaling molecules (autoinducers) that help the microbial cells on the evaluation of their external environment and their inner physiological state, affording the modulation of their populations. We can anticipate that the combination *Ro*:*La*:*Ca* at ratio 1/6:1/6:2/3 inhibits the biofilm formation by the tested bacteria, interrupting their QS system. 

## 3. Materials and Methods

### 3.1. Plant Material 

The samples used in this work come from different sources. The spontaneous species *R. officinalis* L. (*Ro*) was collected in the rural municipality of Talsint, situated in Figuig-Morocco, its geographical coordinates are 32°30′36′′ N, 3°27′36′′ W*. L. angustifolia* (*La*) and *C. aurantium* (*Ca*) were harvested in different locations: PAM garden-Oulmas (33°25′29.3659′′ N, 6°0′5.9002′′ W) and Botanical Garden of the Faculty of Science and Technology (Fes, Morocco), Morocco, respectively. All samples were harvested during the same period (May and June 2018). The used aerial parts (size: 60–100 cm) and leaves (size: 5–10 cm) of *Ro* and *Ca*, respectively, and inflorescences (size: 2–4 cm) of *La* were collected and dried at room temperature (26 at 30 °C) in the dark and then the moisture content (%) of the plant was determined based on the oven method at 100 °C during 24 h.

### 3.2. Hydrodistillation and Simultaneous Hydrodistillation–Steam Distillation

The essential oil extraction was carried out according to the protocol described by El Kharraf et al. [24], using a Clevenger-type and an extractor apparatus. The total weight of plants was 100 g combined in different ratios and distributed (Table 1) in an apparatus as depicted in Figure 1. In the extractor, the two plants were arranged in two layers in a perforated plate, *Ro* in the middle and *La* in the summit, whereas in the round bottom flask, the citrus was submerged in water. In addition, 100 g of *Ro*, *La*, and *Ca* were extracted by conventional hydrodistillation using a Clevenger-type apparatus. During the assays, the same order of plants was always maintained inside the apparatus, as shown in Table 1, and only the plants’ ratios were modified, the final weight being fixed at 100 g. The total volume of water was 1000 mL, and the extraction time was 3 h, the moisture content of the dried plant was 17%. The EOs were stored at 4 °C until use in the upcoming experiments.

### 3.3. Chemical Composition of the Essential Dils:GC-FID/MS Analysis

The GC analysis was performed on a Hewlett Packard (HP 6890) gas chromatograph, equipped with a capillary HP-5 column (30 m × 0.25 mm, film thickness 0.25 µm). The column temperature was programmed from 50 °C after 5 min initial hold to 200 °C with a rate of 4 °C/min. GC-FID/MS was used as previously reported by El-Kharraf [24].

### 3.4. Antioxidant Activity

#### 3.4.1. 2,2-Diphenyl-1-Picrylhydrazyl Free Radical-Scavenging 

The radical scavenging capacity was estimated according to Bounatirou et al. [71] using 2,20-diphenylpicrylhydrazyl (DPPH) free radicals. The radical inhibition percentage was calculated with the equation: Inhibition = [(A_0_ − A_1_)/A_0_ × 100]; A_0_ stands for the absorbance of the control and A1 stands for the absorbance of the sample. The sample concentration providing 50% inhibition (IC_50_) was achieved by plotting the inhibition percentage against the sample concentrations. Tests were carried out in triplicate.

#### 3.4.2. Hydrogen Peroxide Free Radical Scavenging 

The scavenging hydrogen peroxide was evaluated by a colorimetric assay according to Gupta and Sharma [72]. The sample concentration, providing 50% inhibition (IC_50_), was achieved by plotting the inhibition percentage against sample concentrations. Tests were carried out in triplicate.

#### 3.4.3. Nitric Oxide Free Radical-Scavenging Activity

The essential oil NO scavenging activity was estimated using the protocol described by El Guendouz et al. [73] The inhibition percentage was calculated using the formula: Inhibition = [(A_0_ − (A_1_ − A_2_)/A_0_ × 100]. The A_0_ is the absorbance of sodium nitroprusside without sample, A_1_ and A_2_ were the absorbance of the sample with and without sodium nitroprusside.

#### 3.4.4. Superoxide Anion Free Radical Scavenging

Superoxide anion free radical inhibition ability was evaluated using a nonenzymatic PMS-NADH (phenazine methosulfate–nicotinamide adenine dinucleotide) system as previously reported [73]. The inhibition quantification was performed as aforementioned.

### 3.5. Enzymatic Activity

#### 3.5.1. α-Glucosidase Inhibition Activity Assay

The α-glucosidase inhibitor capacity of the essential oils was determined as described by El-Guendouz et al. [73] The crease of absorbance was performed at 405 nm. The inhibition percentage of the enzyme was calculated as follows: I% = [(A_0_ −A_1_)/A_0_) × 100]; where A_0_ is the absorbance of the control and A_1_ is the absorbance of the sample. The trials were triplicated.

#### 3.5.2. Acetylcholinesterase Inhibition Activity Assay

The inhibitory activity of the essential oils on acetylcholinesterase was evaluated according to the method previously described [37]. The following formula was used to determine the percentage of inhibition action: Inhibition = [(A_0_ − A_1_)/A_0_) × 100]; where A_0_ is the absorbance of the control and A_1_ is the absorbance of the sample.

#### 3.5.3. Lipoxygenase Inhibition Activity Assay

The 5-lipoxygenase assay was carried out according to the method described by El Guendouz et al. [73] The following formula was used to determine the percentage of inhibition action: Inhibition = [(A_0_ − A_1_)/A_0_) × 100]; where A_0_ is the absorbance of the control and A_1_ is the absorbance of the sample.

#### 3.5.4. Tyrosinase Inhibition Activity Assay

The tyrosinase inhibition was carried out by the tyrosinase-dependent L-3,4-dihydroxyphenylalanine (l-DOPA) oxidation assay as previously described [37]. The following formula was used to determine the percentage of inhibition action: Inhibition = [(A_0_ − A_1_)/A_0_) × 100]; with A_0_ as the absorbance of the control and A_1_ as the absorbance of the sample.

### 3.6. Antimicrobial Activity

#### 3.6.1. Microorganisms

The Gram negative bacterium *Escherichia coli* DSM 1077, and the Gram positive *Staphylococcus aureus* ATCC 6538 and the methicillin-resistant *S. aureus* 15 (MRSA 15) were used in the present study. The bacterial strains were recovered from −80 °C in the culture medium Brain Heart Infusion (BHI, Oxoid, Basingstock, UK), when required agar (VWR, Radnor, Philadelphia, PA, USA) at 1.5% *w*/*v*. 

#### 3.6.2. Agar Disc Diffusion Method

The bacterial susceptibility to the combinations *Ro:La:Ca* at ratio 1:1:1, *Ro:La:Ca* at ratio 2/3:1/6:16, *Ro:La:Ca* at ratio 1/6:2/3:16, *Ro:La:Ca* at ratio 1/6:1/6:2/3, and the components linalool, 1,8 cineole, and camphor, were performed by the the agar disc diffusion technique as previously described [73]. Briefly, 100 µL of the bacterial suspension at an OD_600nm_ 0.3–0.4 was inoculated in BHI agar, and sterile discs (∅ 6 mm) were distributed over the surface. A volume of 5 µL of each EO and the individual components 1,8-cineole (52.62 mg/mL), and linalool (16.13 mg/mL), were deposited in the center of each disc. The plates were incubated for 24 h. The diameter of the inhibition zone including the disc was measured. The assay was performed in triplicate (n = 6).

#### 3.6.3. Determination of the Minimum Inhibitory Concentration 

The Minimum Inhibitory Concentration (MIC) of the combination *Ro:La:Ca* at ratio 1/6:2/3:1/6 and at ratio 1/6:1/6:2/3, was determined by microdilution. For this, different hydroalcoholic solutions of the samples (10 µL/mL, 25 µL/mL, 50 µL/mL) were prepared in a BHI medium. A volume of 100 µL of BHI supplemented with EOs were distributed onto flat-bottom 96-well microplates. Previously, the bacterial strains were grown in 20 mL of BHI in a shaking water bath overnight at 37 °C at 37 °C. A volume of 100 µL of the overnight bacterial culture diluted in BHI (1:2) were distributed in each well. The incubation was performed at 37 °C, and the growth was followed by spectrophotometry (OD_600 nm_) in a microplate reader (Tecan Infinite, M200, Männedorf, Switzerland). The MIC value was considered as the lowest concentration of the EO that inhibits 95–100 of bacterial growth. Culture medium supplemented with 2-propanol or chloramphenicol (30 µg/mL) were used as control. The Minimum Bactericidal Concentration (MBC) was determined by subculturing from each well. The MBC value was considered the lowest concentration from which it was not possible to recover the bacterial growth. 

#### 3.6.4. Biofilm Formation Inhibition

The inhibition of biofilm formation was done using the crystal violet assay [73]. Briefly, 150 µL of an overnight culture prepared as previously mentioned was centrifuged for 10 min at 4 °C. For the preparation of the bacterial suspension, 150 µL of BHI medium supplemented with EOs at the 2.5 µL/mL, 5 µL/mL and 10 µL/mL, chlorhexidine 0.2% (*v*/*v*), n-propanol (20%), was added to the centrifuged bacterial culture. Each well containing 180 µL of BHI supplemented with EOs at the appropriate concentration was inoculated with 20 µL of the previous bacterial suspension. The cultures with no EO and in the presence of chlorohexidine (0.2 %, *v*/*v*), were used as control. The culture was left to adhere and form a biofilm for 24 h. After this incubation time, non-adherent cells were eliminated and the wells were washed twice with PBS; then, the microplate was left to dry for 15–30 min. Afterwards, crystal violet (4%, *w*/*v*) was added, and after 10 min it was removed. Following this, the wells were washed with PBS. To dissolve the crystal violet, 220 µL of ethanol were added to each well. The absorbance was measured at 595 nm after 15–20 min incubation. 

### 3.7. Statistical Analysis

All assays were carried out in triplicate, and the data are presented as mean ± standard deviation (SD). The independent Tukey’s post hoc and t-student test were used to evaluate for significant differences between group means using the Minitab^®^ 17.1.0 program (LEADTOOLS © 2021-2004, LEAD Technologies, Inc., Charlotte, NC, USA) and GraphPad Prism 9 statistical software. 

## 4. Conclusions

The EO yields of combinations were significantly higher than the individual plants (*Ro*, *La*, and *Ca*) extracted by the conventional hydrodistillation. In the EO of *La*, the major compounds were linalool (21.34%), camphor (14.18%), 1,8-cineole (13.9%), borneol (11.77%), and linalyl acetate (11.58%). The samples of *Ca* EOs showed, as major compounds, linalyl acetate (36.00%) and linalool (30.77%). During the simultaneous hydrodistillation–steam distillation, the steam water and essential vapor from the *Ca* plant in the lower unit of the Clevenger apparatus penetrated the *La* (middle) layer and the *Ro* (summit) layer in the extractor, which may be responsible for the better extraction of some compounds of the EOs.

The antioxidant and enzyme inhibitory activities of EOs cannot be attributed only to their main compounds, since they almost always presented worse activities than the respective essential oils. Moreover, the EOs obtained from two or three plants showed better activity than the single EOs, as observed for the EO obtained by the combination of *Ca* and *Ro*, which possessed a better capacity for scavenging the DPPH free radicals. Other examples include the combination *Ro:La:Ca* (1/6:1/6:2/3) EO, and *Ro:La:Ca* (1/3:1/3:1/3), which showed the greatest scavenging nitric oxide radicals and superoxide anion radicals activities.

Better enzyme inhibitory activities of EO combinations were also observed, as exhibited by the sample *Ro:La:Ca* (2/3:1/6:1/6), which displayed the best inhibitory activity of acetylcholinesterase, close even to the positive control galantamine. The capacity of all EOs for inhibiting the tyrosinase activity was poor, even for the EO combinations.

The combination *Ro:La:Ca* at the ratio 1/6:1/6:2/3 showed the lowest MIC and MBC values against Gram negative and Gram positive strains, which could be related to the enrichment of this combination with 1,8-cineole, linalool, and linalyl acetate. The use of this combination at the mentioned ratio was very efficient in inhibiting the bacterial biofilm formation, even at sub-inhibitory concentrations (1/2MIC value), which suggests that the bacterial QS system was affected.

A combination of EOs isolated from diverse species through one single extraction process (e.g., simultaneous hydrodistillation–steam distillation) improved the biological properties already known for each EO, and with a lower consumption of energy, since only one extraction was required.

## Figures and Tables

**Table 1:** Essential oil yields obtained from the simultaneous hydrodistillation–steam distillation.

*Ro* (Summit)	*La* (Middle)	*Ca* (Lower)	Yields (mL/100 g of Plants)	Color	Odor
1.00	0	0	1.50 ± 0.13 *^c,d^*	colorless	woodsy
0	1.00	0	1.23 ± 0.11 *^e^*	colorless	floral-herbaceous
0	0	1.00	0.76 ± 0.17 *^g^*	greenish	herbaceous
0.50	0.50	0	2.26 ± 0.04 *^a^*	colorless	heavy floral–herbaceous
0	0.50	0.50	1.9 ± 0.10 *^b^*	light yellow	heavy floral–herbaceous
0.50	0	0.50	1.35 ± 0.05 *^f^*	light yellow	herbaceous, strong citrus
0.33	0.33	0.33	1.60 ± 0.10 *^c^*	light yellow	herbaceous, strong citrus
0.67	0.16	0.16	1.50 ± 0.10 *^c,d^*	colorless	woodsy
0.16	0.67	0.16	1.65 ± 0.05 *^c^*	colorless	Floral–herbaceous
0.16	0.16	0.67	1.60 ± 0.10 *^c^*	greenish	herbaceous, strong citrus

Data with different superscript letter are significantly different (*p* < 0.05).

**Table 2:** Chemical composition in percentage of *R. officinalis* (*Ro*), *L. angustifolia* (*La*), *C. aurantium* (*Ca*) EOs and their combinations analyzed by GC-MS.

			Peak Area (%)								
Compounds	RI	*Ro*	*La*	*Ca*	*Ro:La* (1:1)	*Ro:Ca* (1:1)	*Ca:La* (1:1)	*Ro:La:Ca* (1:1:1)	*Ro:La:Ca* (2/3:1/6:1/6)	*Ro:La:Ca* (1/6:2/3:1/6)	*Ro:La:Ca* (1/6:1/6:2/3)
Monoterpene Hydrocarbons											
α-Pinene	939	13.83	tr	nd	tr	tr	nd	tr	tr	tr	tr
Camphene	953	3.82	0.67	nd	2.40	3.11	nd	2.11	3.39	1.78	1.35
β-Pinene	980	2.37	0.64	nd	1.86	3.17	0.24	2.17	2.92	1.64	1.56
β-Myrcene	991	0.63	nd	nd	0.41	0.80	0.26	0.72	0.69	0.96	0.30
Limonene	1020	nd	nd	1.78	nd	0.3	0.49	0.73	nd	nd	1.19
Aromatic Monoterpene Hydrocarbon											
*p*-Cymene	1026	1.26	0.22	nd	1.11	1.45	nd	1.33	1.77	1.28	nd
Oxygenated Monoterpenes											
1,8-Cineole	1033	46.71	13.9	tr	37.81	30.9	11.14	27.77	38.84	16.35	24.27
*cis*-Linalool oxide	1074	nd	3.06	nd	1.41	nd	1.01	0.67	nd	2.28	0.79
*trans*-Linalool oxide	1088	nd	2.48	nd	1.19	nd	1.09	0.59	nd	2.80	0.93
Linalool	1098	nd	21.34	30.77	tr	tr	19.33	12.99	tr	14.02	16.13
Camphor	1143	13.07	14.18	tr	15.14	10.71	11.36	12.65	13.41	12.30	tr
Borneol	1165	tr	11.77	nd	tr	tr	tr	tr	tr	tr	tr
Terpinen-4-ol	1177	0.79	0.51	nd	1.04	1.00	0.47	0.98	1.21	nd	0.50
α-Terpineol	1185	tr	nd	14.97	tr	tr	tr	tr	tr	tr	tr
Linalyl acetate	1261	nd	11.58	36.00	tr	10.71	23.54	11.20	tr	11.48	19.60
Neryl acetate	1365	nd	nd	4.25	nd	0.47	2.01	0.76	nd	0.23	0.32
Geranyl acetate	1383	nd	nd	8.05	nd	nd	3.63	0.36	nd	1.68	0.96
Sesquiterpene Hydrocarbons											
Caryophyllene	1467	6.72	1.70	0.70	0.45	0.84	1.44	0.60	0.76	1.03	0.29
(+)-Epi-bicyclosesquiphellandrene	1483	nd	4.06	nd	1.62	nd	2.97	1.45	0.61	3.05	0.84
Total (%)		98.5	86.59	9.08	97.4	90.44	94.6	94.96	97.8	89.95	94.18

RI: Retention index, nd—not detected, tr—identified components percentage (≤0.1).

**Table 3:** Antioxidant activity of sample EOs extracted by hydrodistillation and simultaneous hydrodistillation–steam distillation.

**Methods**	**Plants Material Ratios**			**Half Maximal Inhibitory Concentration IC_50_ (mg/mL)**			
	***Ro***	***La***	***Ca***	**DPPH**	**H_2_O_2_**	**NO^.^**	**Superoxide**
	**(Summit)**	**(Middle)**	**(Lower)**				
	0	1.00	0	9.01 ± 0.00 *^c^*	26.20 ± 2.77 ^*b*^	ND	2.19 ± 0.58 *^b,c,d,e^*
	0	0	1.00	1.91 ± 0.00 ^*e,d*^	39.62 ± 3.56 *^a^*	0.26 ± 0.02 *^h,i^*	2.94 ± 0.03 *^b,c,d,e^*
Simultaneous Hydrodistillation–Steam Distillation	1/2	1/2	0	2.02 ± 0.02 *^e,d^*	ND	4.11 ± 0.19 *^a,b^*	ND
	1/2 (middle)	0	1/2	0.57 ± 0.09 *^h,j,j^*	ND	1.56 ± 0.18 *^c,d,e,f,g^*	ND
	0	1/2	1/2	0.90 ± 0.07 *^f,g,h,i,j^*	ND	1.45 ± 0.03 *^d,e,f^*	2.47 ± 0.22 *^b,c,d,e^*
	1/3	1/3	1/3	2.34 ± 0.00 *^d^*	28.13 ± 4.14 ^*b*^	2.69 ± 0.23 *^b,c,d^*	10.36 ± 0.73 *^a^*
	2/3	1/6	1/6	1.38 ± 0.02 *^f,g,h^*	ND	1.66 ± 0.05 *^d,e,f^*	ND
	1/6	2/3	1/6	1.37 ± 0.03 *^f,g,h^*	ND	1.63 ± 0.05 *^d,e,f^*	2.98 ± 0.07 *^b,c,d,e^*
	1/6	1/6	2/3	0.75 ± 0.02 *^h,j,i^*	ND	1.12 ± 0.03 *^g^*	1.39 ± 0.02 *^f^*
Major Components	α-Pinene			ND	---	ND	ND
	Limonene			ND	ND	ND	ND
	Borneol			ND	---	ND	ND
	1,8-Cineole			ND	ND	---	ND
	Camphor			ND	---	---	---
	Linalool			102.34 ± 3.13 *^b^*	ND	---	20.41 ± 1.47 *^b^*
	Linalyl acetate			148.61 ± 1.28 ^*a*^	---	---	18.23 ± 0.01 *^b^*
	α-Terpineol			ND	---	ND	ND

Summit, middle, and lower:—plant distribution in simultaneous hydrodistillation–steam distillation. Data represent the mean ± Standard Deviation of triplicate determinations. ND: not determined. ---: no activity. Values in the same column followed by the same letter are not significantly different (*p* < 0.05) by Tukey’s multiple range test.

**Table 4:** Enzymatic inhibitory activity of sample EOs extracted by hydrodistillation and simultaneous hydrodistillation–steam distillation.

Methods	Plant Material Atios			Half Maximal Inhibitory Concentration IC_50_ (mg/mL)			
	*Ro*	*La*	*Ca*	α-Glucosidase	Acetylcholinesterase	Lipoxygenase	Tyrosinase
	(Summit)	(Middle)	(Lower)				
Hydrodistillation	1.00	0	0	0.50 ± 0.07 *^c^*	^†^	^‡^	ND
	0	1.00	0	0.05 ± 0.00 *^i,j^*	0.44 ± 0.00 *^c^*	0.28 ± 0.00 *^c,d^*	ND
	0	0	1.00	0.16 ± 0.00 *^f^*	2.87 ± 0.38 *^a^*	0.18 ± 0.00 *^e^*	ND
Simultaneous Hydrodistillation-Steam Distillation	1/2	1/2	0	0.29 ± 0.01 *^e^*	0.05 ± 0.00 *^e,f,g,h,i,j^*	0.48 ± 0.00 *^a,b^*	ND
	1/2	0	1/2	0.11 ± 0.00 *^g,h^*	0.07 ± 0.0 *^e,f,g,h,i,j^*	0.29 ± 0.00 *^c,d^*	ND
	0	1/2	1/2	0.08 ± 0.00 *^h,i^*	0.06 ± 0.00 *^e,f,g,h,i,j^*	0.44 ± 0.00 *^b,c^*	ND
	1/3	1/3	1/3	0.36 ± 0.01 *^d^*	0.08 ± 0.00 *^e,f,g,h,i^*	0.26 ± 0.00 *^c,d^*	ND
	2/3	1/6	1/6	0.59 ± 0.03 *^c^*	0.03 ± 0.00 *^h,i,j^*	0.35 ± 0.02 *^c^*	ND
	1/6	2/3	1/6	0.05 ± 0.00 *^i,^*^j^	0.08 ± 0.00 *^e,f,g,h,i^*	0.39 ± 0.01 *^b,c^*	ND
	1/6	1/6	2/3	0.74 ± 0.02 *^b,c^*	0.08 ± 0.00 *^e,f,g,h,i^*	0.48 ± 0.01 ^*a,b*^	ND
Major Components	α-Pinene			0.88 ± 0.03 *^b,c^*	---	ND	ND
	Limonene			1.18 ± 0.06 *^a^*	0.08 ± 0.00 *^e,f,g,h,i^*	0.11 ± 0.01 *^f^*	ND
	Borneol			1.14 ± 0.03 *^a^*	0.48 ± 0.05 ^b,c^	ND	ND
	1,8-Cineole			1.59 ± 0.07 *^a,b^*	0.18 ± 0.01 *^d^*	ND	ND
	Camphor			0.73 ± 0.07 *^b,c^*	---	ND	ND
	Linalool			---	---	---	ND
	Linalyl acetate			---	---	0.57 ± 0.03 *^a^*	ND
	α-Terpineol			---	---	ND	ND
Standard	Acarbose			0.014 ± 0.00 *^k,i,j^*	ND	ND	ND

Summit, middle, and lower: plant distribution in simultaneous hydrodistillation–steam distillation. Data represent the mean ± SD of triplicate measurement, ND—not determined, --- no activity, values in the same column followed by the same letter are not significantly different (*p* < 0.05) by Tukey’s multiple range test. ^†^ 0.34 mg/mL, ^‡^ 0.54 mg/m [29].

**Table 5:** Susceptibility of bacterial strains used to the EOs using agar diffusion method, and the determined Minimum inhibitory (MIC) and Minimum Bactericidal Concentrations (MBC) of EOs obtained by simultaneous hydrodistillation–steam distillation of the combination *R. officinalis (Ro)*, *L. angustifolia (La)*, and *C. aurantium* (*Ca*).

Plant Material Ratios			Inhibition Zone (mm)			MIC (μL/mL)			MBC ((μL /mL)		
*Ro* (Summit)	*La* (Middle)	*Ca* (Lower)	*S. aureus* ATCC 6538	Methicillin Resistant *S. aureus* 15	*E. coli* DSM 1077	*S. aureus* ATCC 6538	Methicillin Resistant *S. aureus* 15	*E. coli* DSM 1077	*S. aureus* ATCC 6538	Methicillin Resistant *S. aureus* 15	*E. coli* DSM 1077
1/3	1/3	1/3	15.33 ± 0.63 *^b^*	11.83 ± 0.23 *^b,c^*	11.16 ± 0.23 *^d^*	ND	ND	ND	ND	ND	ND
2/3	1/6	1/6	20.00 ± 0.40 *^a^*	11.16 ± 1.24 *^b,c^*	13.66 ± 1.24^*b*^	ND	ND	ND	ND	ND	ND
1/6	2/3	1/6	20.83 ± 0.84 ^*a*^	12.33 ± 0.23 *^b^*	11.67 ± 2.46 *^d^*	10	75	10	25	125	25
1/6	1/6	2/3	24.00 ± 0.70 ^*a*^	18.33 ± 1.24 *^a^*	12.00 ± 0.81 *^b,c^*	<10	<10	<10	<10	10	<10
1,8-Cineole			10.5 ± 0.70 *^c^*	12.16 ± 0.47 *^b^*	12.16 ± 0.62 *^b,c^*	ND	ND	ND	ND	ND	ND
Camphor			10.5 ± 1.22 *^c^*	11.67 ± 1.02 *^b,c^*	13.00 ± 0.40 *^b^*	ND	ND	ND	ND	ND	ND
Linalool			12.83 ± 0.47 *^b,c^*	13.83 ± 1.24 *^b^*	15.67 ± 0.23 *^a^*	ND	ND	ND	ND	ND	ND
Chloramphenicol (30 μg/mL)			10.16 ± 0.70 *^c^*	6.00 ± 0.00 *^d^*	10.83 ± 0.47 *^d,e^*	ND	30.00	ND	ND	ND	ND

Data represent mean ± standard deviation. ND—not determined. Values in the same column followed by the same superscript letter are not significantly different (*p* > 0.05) by Tukey’s test.

## Data Availability

Not available.

## Sample Availability

Samples of the compounds are not available from the authors.
